# Detection of Natural* Toxoplasma gondii* Infection in Chicken in Thika Region of Kenya Using Nested Polymerase Chain Reaction

**DOI:** 10.1155/2016/7589278

**Published:** 2016-11-17

**Authors:** John Mokua Mose, John Maina Kagira, Simon Muturi Karanja, Maina Ngotho, David Muchina Kamau, Adele Nyambura Njuguna, Naomi Wangari Maina

**Affiliations:** ^1^Department of Medical Laboratory Science, School of Medicine and Health Sciences, Kenya Methodist University, P.O. Box 45240, Nairobi 00100, Kenya; ^2^Department of Public Health, JKUAT, P.O. Box 62000, Nairobi 00200, Kenya; ^3^Department of Animal Sciences, Jomo Kenyatta University of Agriculture and Technology (JKUAT), P.O. Box 62000, Nairobi 00200, Kenya; ^4^Department of Animal Health and Production, Mount Kenya University, P.O. Box 342, Thika 01000, Kenya; ^5^Department of Biochemistry, JKUAT, P.O. Box 62000, Nairobi 00200, Kenya

## Abstract

The detection of* Toxoplasma gondii *in free-range chickens is a good indicator of possible risk to human beings. The aim of this study was to investigate the occurrence of* T. gondii* in free-range chicken using polymerase chain reaction (PCR). Brain samples from 105 free-range chickens from three administrative areas in Thika region, Kenya, were collected, DNA-extracted, and analyzed using PCR to detect presence of* T. gondii*. The overall prevalence of* T. gondii* in all the three areas was 79.0% (95% CI: 70.0–86.4%) and the prevalence across the three areas was not significantly different (*P* = 0.5088; *χ* ^2^ = 1.354). Female chickens had higher (79.4%) prevalence than males (78.6%), although the difference was not significant (*P* = 0.922, *χ* ^2^ = 0.01). However, chickens that were more than 2 years old had significantly (*P* = 0.003; *χ* ^2^ = 11.87) higher prevalence compared to younger ones. The study indicates that there was a high occurrence of* T. gondii* infection in free-range chickens from Thika region and that the infection rate is age dependent. Further studies should be carried out to determine the possible role of roaming chickens in the epidemiology of the disease among humans in the area.

## 1. Introduction

Toxoplasmosis is a zoonosis of increasing importance in both developed and developing countries. It is caused by a protozoan parasite* Toxoplasma gondii* whose main definitive host is the domestic cat while all warm blooded animals are the intermediate hosts. The disease accounts for the highest human prevalence among the parasitic zoonoses [1, 2]. In Kenya, the prevalence of toxoplasmosis in human ranges from 23% to 60% [3, 4]. Humans become infected postnatally by ingesting tissue cysts from undercooked meat, consuming food or drink contaminated with oocysts, or accidentally ingesting oocysts from the environment [5].* Toxoplasma gondii* is known to cause congenital disease and has been implicated as leading cause of meningoencephalitis in patients having HIV/AIDS [6].

In Kenya, most of the chickens are reared under the extensive production system. Free-range poultry meat is popular in Kenya because it is assumed to be healthier than that of caged birds. Anecdotally, some communities recommend the meat to pregnant women in order to deliver healthy babies [7]. The keeping of poultry in highly populated areas increases the risk for transmission of zoonoses and a recent study involving farmers in Thika region, Kenya, has established the risk factors associated with the transmission of toxoplasmosis [7]. Poultry that feed directly from the ground, such as free-range chickens, are exposed to contamination and may serve as indicators of the presence of the parasite in the environment and as a source of infection for other animals including man [8]. In Thika region, farmers indicated that they disposed cat faeces in areas accessible by free-range chickens [7]. In the same region, a higher prevalence (39%) of toxoplasmosis was observed in chicken slaughterhouse workers compared to workers in other types of slaughterhouses [9] and this shows that chicken meat and offal could be a source of the* Toxoplasma-*derived tissue cysts for man.

The definitive diagnosis of* T. gondii* infection is mainly established by parasitological, immunological, and molecular tests. Most studies of* T. gondii* in chickens use mouse bioassays for diagnosis, but this procedure takes long time to become complete and the use of animals is an expensive procedure that involves ethical issues [10]. Molecular methods such as PCR on the other hand have been shown to be more sensitive, specific and take less time compared to other assays. The aim of this study was to determine the prevalence of* T. gondii* infection in free-range chicken in Thika region of Kenya using the nested polymerase chain reaction (nPCR) assay.

## 2. Materials and Methods

### 2.1. Study Area

Thika region (1°4′60S) (37°4′60E) in central Kenya occupies an area of 1,960.2 Km^2^ and has an estimated human population of 864,509 (KNBS, 2009). The district has tropical climate with an annual rainfall ranging between 500 and 1500 mm while the mean temperature is 19.8°C [11]. Majority of farmers in Thika District are small holder-farmers, practicing mixed agriculture, including livestock production, food, and cash crops. Most households in the region have cats kept as pets and for control of rodents. In addition, 60% of the farmers keep free-range chickens [7]. Specimens from chickens were processed and analyzed in laboratories within the Jomo Kenyatta University of Agriculture and Technology (JKUAT) and the Institute of Primate Research (IPR).

### 2.2. Collection and Processing of Brain Samples from Chicken

A cross-sectional study was undertaken in Thika region between February and April 2014. A total of 105 free-range chickens aged between one and 4 years were purchased from households within Kakuzi Subcounty (55), Gatanga Subcounty (20), and Thika Municipality Subcounty (30) of Thika region. Gatanga and Kakuzi Subcounties are rural while Thika Municipality has an urban and a semiurban transition. The sample size per subcounty was dependent on population of cats as informed by the local veterinary officers. Farmer-households were systematically chosen by initially randomizing a starting point. A skip interval of at least 500 meters apart between households was used to choose the next household. The veterinary extension officers in the region helped in the households' selection process.

The purchased free-range chickens were of both sexes and were mainly kept by farmers as a source of meat and eggs. Based on the information provided by the farmers, the chickens were grouped into three groups: >1 year but <1.5 years; ≥1.5 years but <2 years, and ≥2 years. They were sacrificed by a registered veterinary laboratory technician by cervical dislocation [12]. The samples of the head portion of each chicken were placed in separate DNase-free nylon bags, marked appropriately, and later transported in a cool box to the laboratory. Whole brains were then obtained from each chicken head under sterile conditions and kept at −20°C until DNA was extracted.

### 2.3. Extraction of DNA

Genomic DNA was extracted from brain samples using commercial DNA extraction kit (Zymo Research Quick-gDNA™ Miniprep Kit, USA). From each sample, 100 *μ*L of DNA was eluted and stored at −20°C in DNase-free Eppendorf tubes until use. Reference* T. gondii *(RH) DNA was donated by Friedrich-Loeffler-Institut Bundesforschungsinstitut für Tiergesundheit in Germany, through the assistance of Dr. Gereon Schares.

### 2.4. Nested Polymerase Chain Reaction for Detection of* Toxoplasma gondii*

Nested PCR reaction targeting a repetitive 529 bp DNA fragment sequence (GenBank Accession number AF146527) was performed as previously described [13]. The first round of 529 bp nested PCR amplification contained 12.5 *μ*L 2x Taq PCR Master Mix (0.1 U Taq Polymerase, 500 *μ*M DNTPS each, 20 mM Tris-HCl, pH 8.3, 100 mM KCl, and 3 Mm MgCl2), 0.5 *μ*L of the 5 *μ*M primers NF1 and NR1 (Table 1), 1 *μ*L of extracted DNA, and 12.5 *μ*L nuclease-free water. Reactions were cycled 30 times by initial denaturation at 94°C for 2 min, denaturation at 94°C for 1 min, followed by annealing at 58°C for 30 s, an extension step at 72°C for 40 s, and a final extension step at 72°C for 5 minutes. The first round product was diluted 1 : 100. The second round of PCR mixtures contained 1 *μ*L diluted product, 12.5 *μ*L 2x Taq PCR Master Mix, 0.5 *μ*L of each 5 *μ*M primer NF2 and primer NR2 (Table 1), and 10.5 *μ*L nuclease-free water. The second round PCR was cycled 31 times by initial denaturation at 94°C for 2 minutes, denaturation at 94°C for 1 min, followed by annealing at 52°C for 15 s, and a final extension step at 72°C for 20 s. Amplifications were performed using a thermal cycler (Mastercycler Gradient Eppendorf Germany).

The reference* T. gondii *(RH) DNA was used as positive control, while PCR water was used as the negative control. A 100 bp DNA ladder (Invitrogen, Carlsbad, CA, USA) was used as a marker. The two controls were included in every amplification step. The product generated in the second amplification was run in 1.5% agarose gel prestained with 3 *μ*L of ethidium bromide (1 *μ*g/mL) and visualized under ultraviolet (UV) light.

### 2.5. Ethical Consideration

Prior to commencement of the study, all protocols and procedures used were reviewed and approved by the Institute of Primate Research Institutional Animal Care and Use Committee (Approval number: IRC/21/11).

### 2.6. Statistical Analysis

Data were entered into a Microsoft Excel (Microsoft, USA) spreadsheet before being exported to a Statview® package (SAS Institute Inc., Cary, North Carolina, USA) where statistical analyses were performed. For quantitative variables, descriptive statistics were used and frequencies and proportions computed and their corresponding 95% confidence intervals (95% CI) calculated [14] whereas Chi-square test was applied to determine whether there were significant differences between occurrence of the disease and area of origin, sex, and age of the chickens.

## 3. Results

**Figure 1:**
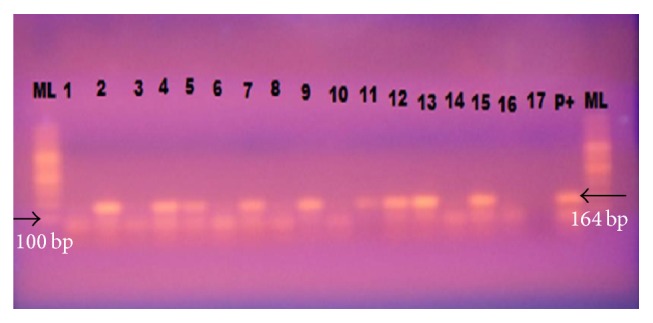
Representative gel photograph for secondary PCR amplification products of* T. gondii* in brain tissues of chicken from Thika region, Kenya. Lanes 1–16: chicken samples; ML: 100 bp DNA ladder.

In this study, presence of* T. gondii *was investigated in a total of 105 chicken brain tissues obtained from Thika region in Kenya, by testing for detection of the 529 bp repeat element. The secondary amplification products clearly showed the predicted amplicon size of 164 bp as shown in Figure 1.

The overall mean prevalence of* T. gondii* in all the three areas was 79.0% (95% CI: 70.0–86.4%). Results of the spatial distribution of* T. gondii* showed that 83.3% (95% CI: 65.3–94.4%) of the chicken brain tissues from Thika Municipality were positive for* T. gondii* (Table 2). The* T. gondii* prevalence for Kakuzi and Gatanga Subcounties was 80.0% (95% CI: 67.0–89.6%) and 70.0% (95% CI: 45.7–88.1%), respectively (Table 2). However, there was no significant (*P* = 0.5088; *χ* ^2^ = 1.354) difference in prevalence of* T. gondii* infection among chicken from the three subcounties.

The chickens sampled in the study were grouped into three groups (>1 year but <1.5 years; ≥1.5 years but <2 years; and ≥2 years) based upon the age (Table 2). Chickens aged 1 year but <1.5 years showed the lowest prevalence (40%) followed by those in age groups ≥1.5 years but <2 years and ≥2 years, respectively. Statistical analysis indicated that the prevalence of* T. gondii* was significantly (*P* = 0.003; *χ* ^2^ = 11.87) associated with age of the bird.

The prevalence of* T. gondii *was apparently higher in females (79.4%) compared to males (78.6%), though the differences were not significant (*P* = 0.922; *χ* ^2^ = 0.01).

## 4. Discussion

This study is the first to report the prevalence of* T. gondii* in chicken, in Kenya. The study was undertaken in an area which was previously shown to have high prevalence of* T. gondii* among the slaughterhouse workers [9]. The high (79%) prevalence of* T. gondii *in all the areas showed that the free-range chickens are a major reservoir for* T. gondii* parasites. The free-range chicken in the study area had free access to habitats around homesteads where they scavenged for feed which mainly included left overs, grass, and insects. As observed by Ogendi et al. [7], most of the homesteads have free-range cats which defecate in the vegetation that the chicken feed on. The latter could explain the high prevalence observed in the study area. In most developing countries, the free-range chickens are slaughtered at home or in unsupervised slaughterhouses and their viscera such as heads are left for scavengers that can include cats and other chicken [15]. The latter allows the lifecycle to be completed in both chicken (intermediate hosts) and cats (definitive hosts). Chickens have been identified as important sources of* T. gondii* in other countries [16] and they serve as important indicator of the potential risk of transmission to human.

The results of this study are close to that of a study done in Brazil where* T. gondii* DNA was amplified in 84.6% of the chicken brain tissue samples and 80.8% of heart samples evaluated [17]. However, the prevalence was higher than that reported in chicken from Brazil (42%) and Egypt (47%) [18, 19].

In the current study, the differences in the prevalence according to area of origin of the chicken may be related to differences in cat densities, the number of chickens examined and sanitation condition in these areas [7]. The higher prevalence of* Toxoplasma* infection in Thika Municipality could be as a result of urban area and periurban livestock keeping and characterized by clustered dwellings, high population density, and poor sewerage system. Kakuzi on the other hand has reported high number of cats around the farms, which is very important, as cats are reservoirs for animal and human toxoplasmosis [7].

A significant relationship between the prevalence of* T. gondii* and the different age groups of chicken was detected in this study. The highest prevalence was detected in older chicken (>2 years) whereas the group of relatively younger chicken (>1–<1.5 years) revealed the lowest prevalence. This direct correlation between the prevalence of* T. gondii* with age of the chicken might be related to the fact that as birds became older, their cumulative likelihood for exposure increased or older birds have had more opportunities to get infection than the younger ones [20, 21].

Female chicken had higher prevalence than males and these results agree with those reported by Akhtar et al. [21]. The variation in prevalence could also be attributed to the fact that female animals are reported to be more susceptible to protozoan parasites as compared to male [22]. A previous report had shown female mice to be more sensitive to pathogenic symptoms of toxoplasmosis than male [23]. However, other reports have reported a higher prevalence rate in males as compared to females [24]. The differences in the hormonal profiles of males and females may play an important role in determining the susceptibility to parasitic infections [25, 26]. Estrogen has been shown to enhance antibody production but immunity can be broken down by various factors including nutrition, age, and reproductive and environmental factors [24].

For a better understanding of the epidemiology and dynamics of* T. gondii* transmission among the various host population, the specific diagnosis of* T. gondii* infections in chickens is important. Previous studies have used serological methods which are characterized by long and laborious test procedures and low sensitivity due to low antibody levels [27]. Mouse bioassays take longer time to diagnose and require ethical considerations [17]. In this study, detection of* T. gondii* DNA in brain samples was done by nPCR, based on the multilocus 529 bp repeat element which gives increased diagnostic sensitivity and accuracy compared to that which can be obtained when targeting the B1 gene that exists in 35 copies/genome [13, 28]. However, the sensitivity of PCR may be limited by the random distribution of the parasite and varying parasite densities in affected tissue [9, 29]. The use of PCR may also be limited by the need for thermocycler, expensive reagents, and skilled manpower. It would be important to investigate the feasibility of using other less expensive, field friendly molecular techniques such as LAMP.

## 5. Conclusions 

The results of this study indicated high level of* Toxoplasma* infection in free-range chicken in Kenya and this could indicate environmental contamination with* T. gondii *oocysts. This occurrence in chicken is central to a better understanding of epidemiology and dynamics of transmission among the various host population. Further genotyping investigations should be carried to determine if the infecting strains in chickens are similar to those circulating in human beings in the area. The generated information will be important for planning an effective optimal prevention and control programs for toxoplasmosis.

## Figures and Tables

**Table 1:** Sequence of nPCR primer sets targeting the *T. gondii* 529 bp repeat element.

Primer	Sequence (5′ to 3′)
(1) NFI	TGACTCGGGCCCAGCTGCGT
(2) NRI	CTCCTCCCTTCGTCCAAGCCTCC
(3) NF2	AGGGACAGAAGTCGAAGGGG
(4) NR2	GCAGCCAAGCCGGAAACATC

**Table 2:** Prevalence of *Toxoplasma gondii *in Thika region, Kenya, based on areas of origin, sex, and age of chicken.

Risk factor	Positive/total samples	Prevalence (%)	*P* and *χ* ^2^ values
Subcounty of origin			
Thika Municipality	25/30	83.3	*P* = 0.5088 *χ* ^2^ = 1.354
Kakuzi	44/55	80	
Gatanga	14/20	70	
*Overall*	*83/105*	*79*	
Age of chicken			
>1 year <1.5 years	4/10	40	*P* = 0.003 *χ* ^2^ = 11.87
≥1.5 years but <2 years	40/45	89	
≥2 years	39/50	78	
Sex of chicken			
Male	33/42	78.6	*P* = 0.922 *χ* ^2^ = 0.01
Female	50/63	79.4	
